# Lysinuric protein intolerance mimicking *N*-acetylglutamate synthase deficiency in a nine-year-old boy

**DOI:** 10.1016/j.ymgmr.2021.100741

**Published:** 2021-03-13

**Authors:** Sarah Al-Qattan, Caroline Malcolmson, Saadet Mercimek-Andrews

**Affiliations:** a Division of Clinical and Metabolic Genetics, Department of Pediatrics, University of Toronto, The Hospital for Sick Children, Toronto, Ontario, Canada; b Division of Hematology, Department of Pediatrics, University of Toronto, The Hospital for Sick Children, Toronto, Ontario, Canada; c Department of Medical Genetics, University of Alberta, Stollery Children's Hospital, Edmonton, Alberta, Canada

**Keywords:** Lysinuric protein intolerance, Hemophagocytic lymphohistiocytosis, Hyperammonemia, Osteoporosis

## Abstract

We report a 9-year-old boy with lysinuric protein intolerance (LPI). He had developmental delay, short stature, failure to thrive, high-protein food aversion, hypothyroidism, growth hormone deficiency, features of hemophagocytic lymphohistiocytosis (HLH), decreased bone mineral density and multiple thoracic spine compression fractures on X-ray. LPI was suspected, but urine amino acid profile and normal orotic acid did not suggest biochemical diagnosis of LPI. Targeted next generation sequencing panel for HLH (including *SLC7A7*) was organized. Due to elevated glutamine in plasma amino acid analysis, a metabolic consultation was initiated and his asymptomatic post-prandial ammonia was 295 μmol/L. We then suspected *n*-acetylglutamate synthase or carbamoyl-phosphate synthase I deficiency due to marked hyperammonemia, elevated glutamine level, normal orotic acid, and normalization of ammonia at 2 h of carglumic acid (200 mg/kg/d). His targeted next generation sequencing panel for HLH revealed homozygous pathogenic variant in *SLC7A7* ((NM_001126106.2): c.726G>A (p.Trp242*)) and confirmed the diagnosis of LPI. We emphasize the importance of genetic investigations in the diagnosis of LPI.

## Highlights

• LPI associated hyperammonemia responds to carbaglumic acid.
• Protein aversion, and failure to thrive should warrant for ammonia measurement.
• Multisystem disease should include LPI into the differential diagnosis even in the absence of typical biochemical features.

## 1 Introduction

Lysinuric protein intolerance (LPI) (MIM#222700) is an inherited metabolic disease due to biallelic variants in *SLC7A7* (MIM#603593) on chromosome 14q11 [[1], [2], [3], [4], [5]]*. SLC7A7* encodes cationic amino acid transporter y^+^LAT-1 subunit, one of the four systems for the transport of cationic amino acids through plasma membranes including lysine, arginine and ornithine [[1], [2], [3], [4], [5], [6]]. Due to the variants in *SLC7A7,* lysine, arginine and ornithine are excreted in the urine and stool resulting in lysine, arginine and ornithine deficiencies. Lysine deficiency results in connective tissue abnormalities due to abnormal crosslinking of collagen peptides, carnitine deficiency resulting in dysfunction of fatty acid metabolism, abnormal uptake of iron, and protein deficiency [1,2]. Arginine and ornithine deficiencies result in postprandial hyperammonemia and protein aversion due to secondary urea cycle metabolism defect. More than 200 patients with LPI from 25 different countries have been reported worldwide [1].

There is a significant heterogeneity in disease severity including infantile onset failure to thrive after the initiation of solid foods, hypotonia, hepatosplenomegaly, global developmental delay to recurrent fractures secondary to osteoporosis, and adult-onset episodic encephalopathy. Increased excretion of lysine, arginine and ornithine in urine amino acid- analysis is the biochemical hallmark of LPI however it can be masked by protein malnutrition. Postprandial hyperammonemia may also suggest LPI. The diagnosis is confirmed by sequencing of *SLC7A7.*

We report a new patient with LPI who presented with marked asymptomatic hyperammonemia, elevated glutamine, and normal orotic acid and a dramatic response to the first dose of carglumic acid, thereby leading to a suspected diagnosis of *n*-acetylglutamate synthase (NAGS) or carbamoyl-phosphate synthase I (CPS1) deficiencies.

## 2 Patient and results

This 9-year-old boy was born to consanguineous parents (first cousins, and grandparents first cousins) after an unremarkable pregnancy via C-section due to history of malposition. His birth weight was 3000 g (25th percentile). He was exclusively breastfed for the first 8 months of life. He had several upper respiratory tract infections from the age of 4 months. Solid foods were introduced at 8 months of age. He demonstrated a strong aversion to yogurt and cow's milk leading to episodes of vomiting and poor tone. He refused to eat meat, chicken, and fish. His diet mainly consisted of rice, olive oil, bread and other types of carbohydrates. He had four non-bloody bowel movements daily. The family moved to Canada, when he was 8 years old. We summarized his multi-system phenotypes below:

### 2.1 Endocrinological phenotype

**Fig. 1:**
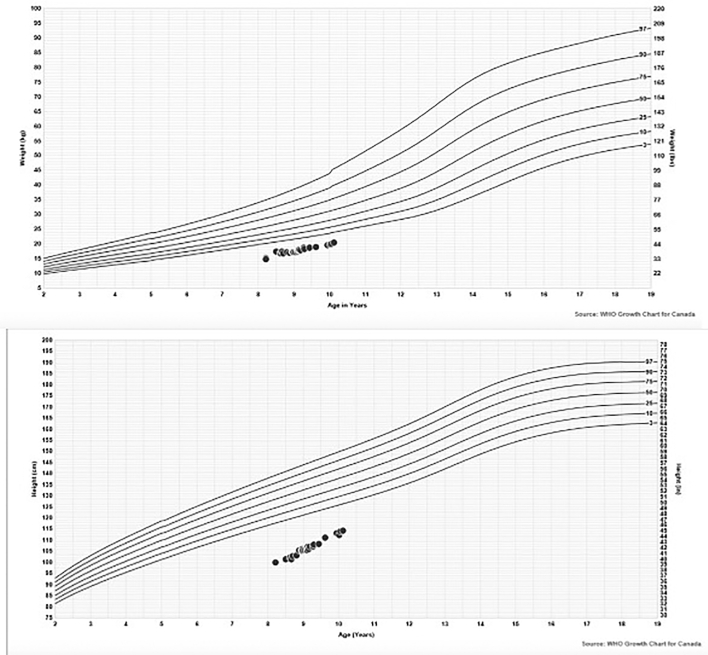
All weight and height measurements are depicted in Fig. 1.

Due to hypothyroidism, he was treated with levothyroxine from the age of 2.5 years. He presented to our institution at 8.5 years of age for the management of his growth hormone and thyroid hormone therapies. His height and weight percentiles are depicted in Fig. 1. He was on 25 μg of levothyroxine and 0.35 mg injections of somatropin daily (corresponding to 0.12 mg/kg/day). His TSH (2.33 mIU/L; reference range: 0.73–4.09 mIU/L) and free T4 (12.3 pmol/L; reference range: 10.0–17.6 pmol/L) were normal. As he had a poor response to the growth hormone stimulation test, his growth hormone dose was increased to 0.45 mg/day of somatropin via subcutaneous injections. This treatment maintained his growth but did not improve his height percentile (Fig. 1).

### 2.2 Hematological phenotype

He had mild anemia, and markedly elevated ferritin (1452.6 μg/L; reference range 13.7–78.8 μg/L) and was referred to hematology clinic. His complete blood count (CBC) revealed a mild normocytic anemia with a hemoglobin of 94 × 10^9^ g/L (reference range 106–134 × 10^9^) and a mean corpuscular volume (MCV) of 80.4 fL (reference range 74.4–86.1). His red blood cell (RBC) morphology revealed polychromasia and occasional schistocytes. He had mildly elevated reticulocytes (76.8 × 10^9^ g/L; reference range 42.4–70.2 × 10^9^), elevated unconjugated bilirubin (14 μmol/L), elevated ferritin (1452.6 μg/; reference range 13.7–78.8), low fibrinogen (1.3 g/L; reference range 1.9–4.3), and elevated LDH (1943 U/L; reference range 420–750 U/L). Direct antiglobulin test was negative and haptoglobin was low normal. His hemoglobin electrophoresis was normal. Peripheral flow for paroxysmal nocturnal hemoglobinuria clones was negative. Incubated osmotic fragility was normal. There was no evidence of pyruvate kinase or G6PD deficiencies. His abdominal ultrasound revealed mild enlargement of liver with normal liver echogenicity and no splenomegaly.

He underwent a bone marrow aspirate and biopsy at age 8 years and 7 months which revealed adequate cellularity, adequate megakaryocytes, normal iron storage, no evidence of ringed sideroblasts, normal erythropoiesis, all stages of granulopoiesis present, and no clear morphological evidence of malignant infiltration. Several histiocytes were noted with evidence of hemophagocytosis. He had three of the eight HLH diagnostic criteria [7] including hypofibrinogenemia, hemophagocytosis in the bone marrow, and an elevated ferritin. Despite he did not meet diagnostic criteria for HLH, he underwent targeted next generation sequencing panel for HLH (including 14 genes).

Coagulation studies revealed a persistently prolonged PT (14.3; reference range 7.9–11.9 s), prolonged INR (1.3 and 1.4; reference range 0.8–1.2) and intermittently prolonged PTT (between 29 and 39; reference range 24–36 s). A 1:1 mixing study showed full correction suggestive of factor deficiency. His factor V level was low (0.37 IU/mL with a reference range of 0.71–1.68 IU/mL). He had normal levels of factors VII (0.77 IU/mL; reference range 0.57–1.59 IU/mL), VIII (1.44 IU/mL; reference range 0.56–1.72 IU/mL), IX (0.63 IU/mL; reference range 0.74–1.66 IU/mL), X (0.75 IU/mL; reference range 0.69–1.54 IU/mL), XI (0.79 IU/mL; reference range 0.63–1.52 IU/mL), and XII (1.69 IU/mL; reference range of 0.40–1.49 IU/mL). Our patient had no history of bruising or bleeding.

### 2.3 Immunological phenotype

Due to persistently elevated EBV titre (120,000 IU/mL), he was referred to immunology clinic. His soluble IL-2 receptor (CD25) was normal (486 U/mL; reference range 278–1580). He had normal natural killer degranulation activity and the expression of perforin protein in CD56 + cells. He had normal T and B cell numbers, normal mitogen stimulation (T cell function), and normal immunoglobulin levels. His immunological functions did not reveal any immunological deficits.

### 2.4 Skeletal phenotype

Chest X-ray showed multiple thoracic spine compression fractures.

### 2.5 Respiratory phenotype

Chest X-ray showed bilateral mild peri-bronchial thickening, a nonspecific lower airway inflammation. There were no reported respiratory symptoms. He did not have respiratory phenotype at the time of his diagnosis.

### 2.6 Renal phenotype

His creatinine was normal as well as his kidney ultrasound. He did not have renal phenotype at the time of his diagnosis.

### 2.7 Metabolic phenotype and diagnosis

He was first seen in our clinic for genetic investigations at age 9 years. Developmentally, he walked independently at age 4 years, spoke in sentences at age 5 years, started feeding himself and dressing himself at age 6 years. In his family history, he had two healthy brothers (14 and 12 years), a pregnancy loss at 6 weeks gestation in his mother, a sister with hydrocephalus, who died in infancy, a paternal uncle with aversion to meat, and a maternal first cousin with developmental delay.

His physical examination revealed dysmorphic features including a square trunk, frontal bossing, long eyelashes, telecanthus, broad nasal root, anteverted nares, bilateral 5th finger clinodactyly, left single palmar crease, and prominent heels. His height and weight were <1st percentile (Fig. 1), and his head circumference was at 33rd percentile. His liver was 3 cm below the costal margin.

Based on his phenotypes, LPI was suspected. Metabolic investigations and targeted next generation sequencing panel for HLH were initiated. All investigations are summarized in Table 1A, Table 1B. His non-fasting plasma amino acid analysis revealed low ornithine, lysine and arginine and elevated alanine and glutamine levels. His non-fasting urine amino acid analysis revealed normal ornithine and lysine levels and mildly elevated arginine level with normal urine specific gravity (1.025; reference range 1.005–1.035). Urine orotic acid was normal. His post-prandial ammonia was 157 μmol/L (reference range <35; urgent repeat 295 μmol/L). Due to normal orotic acid, and elevated glutamine, a proximal urea cycle defect was suspected. He was started on intravenous (IV) fluids (10% dextrose at 1.5 times maintenance). Plasma and urine amino acid analysis were repeated (Table 1A). A repeated ammonia was 326 μmol/L after 3.5 h of IV fluids. Carglumic acid (200 mg/kg/day in three doses) and arginine (250 mg/kg/dose for 1.5 h) were started. At 2 h of the first dose of carglumic acid his ammonia was 46 μmol/L. His ammonia levels were between <9–32 μmol/L for 4 days. He was discharged on carglumic acid (35 mg/kg/day), arginine (86 mg/kg/day), essential amino acid medical formula and protein-restricted diet (1.2–1.5 g/kg/day natural protein; and medical protein to natural protein ratio of 1:5). NAGS deficiency was suspected based on his response to carglumic acid, *NAGS* sequencing was requested.

**Table 1A:** Metabolic investigations of the patient with LPI are summarized in the below table.

	Amino acids & reference ranges	A	B	C	D	E	F	G	H	I
		8 y 9 m	9 y 1 m	9 y 1 m	9 y 1 m	9 y 1 m	9 y 2 m	9 y 3 m	10 y 1 m	10 y 1 m
Plasma amino acids μmol/L	Arginine 66–150	8	5	13	12	13	27	15	14	12
	Lysine 102–259	38	36	99	53	33	42	75	86	88
	Ornithine 34–94	8	9	9	9	13	20	12	15	12
	Asparagine 38–91	135	145	105	93	145	91	70	163	129
	Citrulline 16–41	44	71	56	46	51	98	41	53	45
	Glutamine 467–755	1280	1807	1429	1155	1389	1429	999	1954	1576
	Glutamate 74–266	75	62	83	49	84	32	42	86	136
	Glycine 176–398	550	672	578	578	521	366	354	690	555
	Alanine 208–588	1419	1495	1573	1520	817	384	372	1884	2108
	Proline 118–372	428	615	469	281	402	179	219	529	544
	Serine 112–216	242	317	200	188	269	194	147	339	279
	Aspartate 20–42	4	3	5	3	5	<2	3	5	6
	Valine 128–361	186	197	192	152	194	154	140	229	170
	Leucine 85–226	97	111	83	45	112	48	69	147	68
	Isoleucine 43–129	58	69	53	28	66	39	44	94	40
	Threonine 72–185	192	190	240	174	208	167	118	209	219
Carnitines	Total carnitine 32.0–84.0 μmol/L	28.9	NP	NP	20.9	112.1	NP	82	NP	NP
	Free carnitine 26.0–60.0 μmol/L	25.7	NP	NP	16.6	83.5	NP	59	NP	NP
Lactate	<2.4 mmol/L	NP	1.4	NP	NP	NP	NP	NP	NP	NP
Ammonia	<35 μmol/L	NP	326	32	19	21	<9	<9	93	34
Urine amino acids mmol/mol creatinine	Lysine 4–408	382	1331	NP	NP	511	1448	1648	NP	1293
	Arginine 1–7	22	20,371	NP	NP	38	1552	116	NP	68
	Ornithine 2–57	8	5422	NP	NP	9	197	24	NP	21
	Cystine 3–123	13	481	NP	NP	13	50	25	NP	19
Urine orotic acid mmol/mol creatinine	0.5–3.3	1.2	NP	NP	NP	NP	NP	NP	NP	NP

*A – 8 years and 9 months of age:* The patient with LPI was assessed in the clinical genetics clinic and LPI was suspected. Urine and plasma amino acid analysis did not suggest LPI or any specific inherited metabolic disease. These samples were collected prior to his LPI diagnosis.

*B – 9 years and 1 month of age:* The patient with LPI had his first ammonia measurement which was elevated and was collected prior to his LPI diagnosis.

*C – 9 years and 1 month of age:* The patient with LPI after 4 days of treatment with arginine and carglumic acid and self-restricted protein intake (1–1.2 g/kg/day total protein). These samples were collected prior to LPI diagnosis.

*D – 9 years and 1 month of age:* The patient with LPI at 4 days of arginine (28.7 mg/kg/dose) and carglumic acid (11.5 mg/kg/dose) treatment in addition to protein-restricted diet (1.25 g/kg of natural protein intake) and essential amino acid supplement (0.3 g/kg). These samples were collected prior to LPI diagnosis.

*E – 9 years and 1 month of age:* The patient with LPI assessed 2 weeks after his initial hyperammonemia diagnosis and confirmed genetic diagnosis of LPI. Arginine was discontinued and citrulline (84 mg/kg/day) and carnitine (42 mg/kg/day) were started. He continued on carglumic acid (11.5 mg/kg/dose) and protein-restricted diet (1.25 g/kg of natural protein intake) and essential amino acid supplement (0.3 g/kg). These samples were collected at the time of molecular genetic diagnosis of LPI.

*F – 9 years and 2 months of age:* The patient with LPI had his normal *NAGS* genetic test result after 3 weeks of his initial hyperammonemia diagnosis and discontinuation of carglumic acid for 2 days and protein-restricted diet (1.5 g/kg of natural protein intake) and essential amino acid supplement (0.3 g/kg). These samples were collected after the diagnosis of LPI.

*G – 9 years and 3 months of age:* The patient with LPI was on lysine (50 mg/kg/day), and on the same doses of citrulline and carnitine, protein-restricted diet (1.0 g/kg of natural protein intake) and essential amino acid supplement (0.3 g/kg). These samples were collected after the diagnosis of LPI.

*H – 10 years and 1 month of age:* The patient with LPI was started on sodium phenylbutyrate (225 mg/kg/day) and same doses of lysine, citrulline and carnitine, protein-restricted diet (1.7 g/kg of natural protein intake) and essential amino acid supplement (0.25 g/kg). These samples were collected after the diagnosis of LPI.

*I – 10 years and 1 month of age:* The patient with LPI and his response 4 days after starting sodium phenylbutyrate. These samples were collected after the diagnosis of LPI.

**Table 1B:** Biochemical investigations of the patient with LPI are summarized in the below table. All of these investigations were collected prior to his LPI diagnosis by different clinics.

	Investigations (reference ranges)	Result
**Lipids**	Triglyceride (<0.85 mmol/L)	2.07
**Liver function tests**	AST (<43 U/L)	72
	ALT (<24 U/L)	36
	GGT (<13 U/L)	17
	LDH (420–750 U/L)	3832
	Unconjugated bilirubin (<9 μmol/L)	15
	PT (7.9–11.9 s)	14.3
	INR (0.8–1.2)	1.4
	PTT (24–36 s)	38
	Albumin (37–50 g/L)	44
**Haptoglobin**	0.07–1.63 g/L	<0.08
**Ferritin**	13.7–78.8 μg/L	1452.6
**Zinc**	30–65 μmol ZZP/mol Heme	47
**Iron**	4.8–25.3 μmol/L	19.8
**CBC**	WBC (4.31–11.00 ×10^9^/L)	5.22
	RBC (3.96–5.03 ×10^12^/L)	3.96
	HGB (107–134 g/L)	118
	HCT (0.322–0.398 L/L)	0.319
	PLT (206–369 ×10^9^/L)	227
	MCV (74.4–86.1 fL)	80.6
	Absolute retic count (42.4–70.2 × 10^9^/L)	78
	Plasma hemoglobin (0–29 mg/L)	166
**Growth**	GH post-stimulation (>5.7 μg/L)	1.1
	IGF-1 (60–414 μg/L)	26
**Bone health**	Ionized calcium (1.22–1.37 mmol/L)	1.25
	Phosphate (1.41–2.02 mmol/L)	1.53
	Magnesium (0.70–0.95 mmol/L)	0.76
	ALP (143–318 U/L)	289
	Total 25-OH Vitamin D (70–250 nmol/L)	22
**Thyroid functions**	TSH (0.73–4.09 mIU/L)	1.20
	Free T4 (10.0–17.6 pmol/L)	12.8
**Renal function**	Creatinine (25–50 μmol/L)	25
**Urinalysis**	Protein (negative g/L)	Negative
	Hemoglobin (negative)	Negative

Abbreviations: AST: Aspartate aminotransferase; ALT: Alanine aminotransferase; GGT: Gamma-glutamyl transferase; LDH: Lactate dehydrogenase; PT: Prothrombin time; INR: International normalized ratio; PTT: Partial thromboplastin time; CBC: Complete blood count; WBC: White blood cells; RBC: Red blood cells; HGB: Hemoglobin; HCT: Hematocrit; PLT: Platelets; MCV: Mean corpuscular volume; IGF-1: Insulin-like growth factor 1; TSH: Thyroid stimulating hormone.

His targeted next generation sequencing panel for HLH revealed a homozygous known variant, *SLC7A7* (NM_001126106.2): c.726G>A (p.Trp242*) [5] confirming the diagnosis of LPI. Arginine was discontinued and citrulline (100 mg/kg/d), carnitine (50 mg//kg/day) and lysine (50 mg/kg/day) were started. His genetic testing for NAGS deficiency was negative and carglumic acid treatment was discontinued after 3 weeks of initial therapy start. The post-prandial ammonia levels were between <9–63 μmol/L. He had excellent compliance to the protein-restricted diet and supplemented with medical formula containing essential amino acids. His total protein intake ranged between 1.3 and 1.9 g/kg/day including natural protein intake (range 1.3–1.7 g/kg/day) and medical formula intake (0.3 g/kg/day) (Table 1A). Due to low branched chain amino acids, his natural protein intake was gradually increased which resulted in high post-prandial ammonia and glutamine levels. His non-fasting plasma amino acid analysis showed elevated glutamine levels even with normal ammonia levels. The lowest glutamine level was 999 μmol/L with the lowest natural protein intake of 1 g/kg/d (Table 1A legend). When he had a post-prandial ammonia of 93 μmol/L at 1 year of therapy, sodium phenylbutyrate was started (225 mg/kg/day). Four days later, his post-prandial ammonia was 34 μmol/L.

### 2.7 Investigations performed after the LPI diagnosis

His bone mineral density revealed lowest z-score of −4.8 of total body (reduced bone density) at age 9 years. Neuropsychological assessments were difficult to apply due to significant delays and language barrier at age 9 years 2 months. Details of the neuropsychological assessments are summarized in the supplemental data.

## 3 Discussion

We report a new patient with LPI and his diagnostic odyssey over 9 years, despite he was symptomatic from the first year of life. Low levels of ornithine, arginine and lysine in plasma amino acid analysis, and normal ornithine, lysine, and mildly elevated arginine level in urine amino acid analysis was not suggestive of LPI. His protein intake was 1–1.2 g/kg/day and he was meeting dietary reference intake (age appropriate intake of 0.95 g/kg/day) for protein. Our initial suspicion of LPI was not supported by urine amino acid analysis. His metabolic investigations did not suggest any particular inherited metabolic disorder that present with hyperammonemia. Elevated glutamine level in plasma amino acid analysis, hyperammonemia, and normal orotic acid level raised the suspicion of NAGS or CPS1 deficiency, despite marginal elevation of citrulline level. Due to asymptomatic hyperammonemia and our suspected diagnosis of NAGS or CPS1 deficiency, carbaglumic acid was chosen as a cost effective first-line ammonia scavenger. We did not use the suggested international guidelines for the diagnosis and management of urea cycle disorders [30], as our patient had an atypical presentation . He would likely have shown the same response to IV sodium phenylacetate and sodium benzoate. We think that carglumic acid was quick and cost effective to treat marked hyperammonemia in an asymptomatic patient with LPI. As his postprandial ammonia levels were manageable with diet and supplements up to 1 year of treatment, we did not add sodium benzoate or sodium phenylbutyrate to his treatment until we saw moderate post-prandial hyperammonemia. We did not see any improvements in hyperammonemia on IV glucose. As arginine IV and carglumic acid treatments were started at the same time, it is likely that IV arginine may have contributed almost normal ammonia levels after 2 h of the first dose of carglumic acid. His other phenotypes were not suggestive of NAGS or CPS1 deficiency but were attributed to the possibility of a second genetic disease due to consanguinity of parents and grandparents. His urine amino acid profile was only suggestive of LPI after arginine and lysine supplementations. It is important to note that non-diagnostic urine amino acid analysis and normal urine orotic acid are not sufficient to exclude LPI. Postprandial hyperammonemia in LPI results in intermittent encephalopathy, developmental delay and cognitive dysfunction [2,[8], [9], [10], [11], [12]]. Interestingly, our patient was alert, and oriented, when we identified markedly elevated ammonia for the first time. He had no history of intermittent encephalopathy episodes. In patients with multisystem organ involvement, failure to thrive and aversion to protein intake, LPI should be included into the differential diagnosis.

Emerging use of carglumic acid was reported in the treatment of hyperammonemia for NAGS deficiency, CPS1 deficiency, propionic acidemia, methylmalonic acidemia, isovaleric acidemia, and glutamate dehydrogenase overactivity [13]. *N*-acetylglutamate is a cofactor and synthetized from glutamate and acetyl-CoA by NAGS in the mitochondrial matrix. *N*-acetylglutamate activates CPS1 to synthetize carbamoyl-phosphate from ammonia and bicarbonate in the urea cycle. In the absence of *n*-acetylglutamate due to NAGS deficiency, CPS1 cannot be activated, and ammonia cannot be detoxified. Carbaglu (n-carbamylglutamate) is the synthetic form of *N*-acetylglutamate and activates CPS1 and treats hyperammonemia in NAGS deficiency. In CPS1 deficiency, carglumic acid likely stimulates the residual CPS1 activity and improves partially hyperammonemia. Accumulation of propionyl-CoA inhibits NAGS and decreases *n*-acetylglutamate in organic acidemias and prevents ammonia detoxification. Increased glutamate dehydrogenase activity results in glutamate deficiency due to excessive oxidation of glutamate, which is required for the production of *n*-acetylglutamate. *N*-acetylglutamate deficiency is the result of inactive CPS1 and hyperammonemia. In LPI, the presumed hypothesis of hyperammonemia is arginine and ornithine deficiencies and urea cycle dysfunction, but *n*-acetylglutamate deficiency has not been reported so far. There is no glutamate deficiency as well. To the best of our knowledge, we report successful management of hyperammonemia using carglumic acid in LPI for the first time. Our results may help us to better understand mechanisms of hyperammonemia in LPI which may guide the management of hyperammonemia in the future.

False negative urine lysine, ornithine and arginine excretion secondary to protein malnutrition was reported in LPI [14,15]. Variable phenotypes pose a diagnostic challenge in LPI [4,16,17]. In an epidemiological study, more than half of the patients had protein aversion (94%) hepatomegaly (69%), failure to thrive (64%), intellectual disability (55%), nausea and vomiting (52%) and encephalopathy (52%) [16]. High index of suspicion is important in the absence of typical biochemical features to diagnose LPI. Targeted next generation sequencing panels for HLH, intellectual disability, or short stature or exome sequencing can identify patients with LPI at their symptom onset.

Our patient showed low factor V level, which is involved in the intrinsic and extrinsic coagulation pathways that explains prolongation of intrinsic and extrinsic pathways [17]. The decreased factor V level cannot be explained by hepatic dysfunction as the other coagulation factors were normal. The remaining hematological findings were similar to the patients reported in the literature [9,10,15,16,[18], [19], [20], [21], [22], [23]].

Growth hormone deficiency is secondary to arginine depletion in LPI [2]. Improved growth on long-term growth hormone therapy in LPI was reported in one study [24]. However, another study did not show significant increase in growth velocity on growth hormone therapy in LPI [25]. Our patient has no growth spurt on growth hormone therapy as well. Larger studies are required to evaluate cost effectiveness of growth hormone therapy in LPI.

Pathological fractures secondary to osteoporosis are common in LPI [11,26,27]. Dietary protein restriction and lysine deficiency results in defective matrix protein synthesis and impaired capacity for maintenance of normal bone remodelling [28,29]. We identified diffuse osteoporosis and asymptomatic multiple thoracic spine compression fractures in our patient. A monitoring of bone mineral density will help us to understand, if vitamin D and calcium supplements would improve bone health in LPI.

We report a new patient with LPI, who had asymptomatic marked hyperammonemia and an excellent response to carglumic acid mimicking NAGS deficiency. Initial arginine supplementation may have contributed to normalization of ammonia levels, while on carglumic acid. Arginine and citrulline are intermediary amino acids of the urea cycle and their deficiency results in hyperammonemia, which improves by their supplementation. Nonetheless, we emphasize the importance of targeted next generation sequencing or exome sequencing in the early diagnosis of LPI in patients with multisystem involvement.

The following are the supplementary data related to this article.

Supplemental Data 1 Details of the neuropsychological assessments are summarized in the supplemental data. Supplemental Data 1
