# Supplementary material for: Lysinuric protein intolerance mimicking N-acetylglutamate synthase deficiency in a nine-year-old boy
Source: Mol Genet Metab Rep. 2021 Mar 13;27:100741. doi: 10.1016/j.ymgmr.2021.100741 (PMC7973239; doi:10.1016/j.ymgmr.2021.100741)
Supplement: Supplemental Data 1 — Details of the neuropsychological assessments are summarized in the supplemental data. [file mmc1.docx]

**Lysinuric protein intolerance mimicking N-acetylglutamate synthase deficiency in a nine-year-old boy**

**Supplemental Data**

Neuropsychological assessments were difficult to apply due to significant delays and language barrier at age 9 years 2 months. A battery of neuropsychological tests was administered to assess nonverbal intellectual abilities, early English language development, visual/motor skills, fine motor dexterity/coordination, visual memory, and parent and teacher rated adaptive, emotional, and behavioral functioning. He requires individual education plan and modified curriculum.

Neuropsychological assessments administered are listed below:

1. Beery-Buktenica Developmental Test of Visual-Motor Integration - 6th Edition (VMI)
2. Bracken Basic Concepts Scale - Third Edition (Receptive) (Bracken-3)
3. Children's Memory Scale
4. Expressive Vocabulary Test - Third Edition (EVT-3)
5. Grip Strength
6. Kaufman Assessment Battery for Children - Second Edition (KABC-2)
7. Leiter International Performance Scale - Third Edition
8. NEPSY - 2nd Edition, Selected Subtests
9. Peabody Picture Vocabulary Test - 5th Edition (PPVT-5)
10. Purdue Pegboard Test
11. MVP
12. Parents completed the following questionnaires and standardized rating scales:
13. Vineland Adaptive Behavior Scales - Third Edition (VABS-3)
14. Strengths and Difficulties Questionnaire (SDQ)
15. Teachers completed the following questionnaires and standardized rating scales:
16. Vineland Adaptive Behavior Scales - Third Edition (VABS-3)
17. Strengths and Difficulties Questionnaire (SDQ)

Based on his neuropsychological assessment results:

- Low average to average range long-term recall
- Low average to average adaptive functioning
- Extremely low range self-care skills as per teacher reports
- Extremely low range nonverbal intellectual abilities
- Fine motor skills, fine motor dexterity, motor coordination, and visual-motor integration estimated at about a 3–4-year-old level
- Difficulty in regulating his attention and activity level as per teacher reports
